# Controlled Hydrolysis of Odorants Schiff Bases in Low-Molecular-Weight Gels

**DOI:** 10.3390/ijms23063105

**Published:** 2022-03-13

**Authors:** Gloria Nicastro, Louise Mary Black, Paolo Ravarino, Simone d’Agostino, Davide Faccio, Claudia Tomasini, Demetra Giuri

**Affiliations:** 1 Dipartimento di Chimica Giacomo Ciamician, Università di Bologna, Via Selmi 2, 40126 Bologna, Italy; gloria.nicastro@studio.unibo.it (G.N.); paolo.ravarino2@unibo.it (P.R.); simone.dagostino2@unibo.it (S.d.); davide.faccio2@unibo.it (D.F.); 2 School of Chemistry, University of Glasgow, Glasgow G12 8QQ, UK; louise.black@studio.unibo.it

**Keywords:** Schiff base, hydrolysis, gel, self-assembly, fragrance, perfumery

## Abstract

Imines or Schiff bases (SB) are formed by the condensation of an aldehyde or a ketone with a primary amine, with the removal of a water molecule. Schiff bases are central molecules in several biological processes for their ability to form and cleave by small variation of the medium. We report here the controlled hydrolysis of four SBs that may be applied in the fragrance industry, as they are profragrances all containing odorant molecules: methyl anthranilate as primary amine, and four aldehydes (cyclamal, helional, hydroxycitronellal and triplal) that are very volatile odorants. The SB stability was assessed over time by HPLC-MS in neutral or acidic conditions, both in solution and when trapped in low molecular weight gels. Our results demonstrate that it is possible to control the hydrolysis of the Schiff bases in the gel environment, thus tuning the quantity of aldehyde released and the persistency of the fragrance.

## 1. Introduction

Fragrances are low molecular weight molecules with a characteristic odor [1,2,3]. The high volatility of these compounds can be a problem not only during their storage but also during their release, which is hard to control over time, limiting the persistency of the scent. The perfume industry developed several strategies and release technologies to assure fragrances’ long-lastingness and stability. A possible strategy is to entrap the odorant molecules in micelles, capsules or particles [4,5,6,7] that often have to face problems like low encapsulation load or poor material stability. Another strategy, adopted also by nature for the storage of volatile species, is to create precursors with reduced volatility. This can be obtained by covalently binding the fragrance to another substrate, creating profragrances or properfumes. The covalent bond should be then selectively cleaved by a specific stimulus, such as oxidation, light, enzymes, pH change, heat or hydrolysis, releasing the perfumed molecule [8,9,10,11,12,13].

In this scenario, Schiff bases (SB) represent valid profragrances that can be synthesized starting from odorant aldehydes and primary amines through a condensation reaction [14,15,16]. The reaction is reversible, and the SB can be readily hydrolyzed to the starting materials in aqueous environments, mainly in acidic conditions [17].

In this work, we report the controlled hydrolysis of four SBs that can be used as profragrances in perfumery applications. The SBs are synthesized starting from five odorant species: the primary amine methyl anthranilate (MA; b.p. = 256 °C) [1], combined with four aldehydes: cyclamal (A1; b.p. = 270 °C), helional (A2; b.p. = 282 °C), hydroxycitronellal (A3; b.p. = 262 °C) and triplal (A4; b.p. = 196 °C) [18]. 

The stability of the four SBs was assessed in EtOH/H_2_O solutions, studying the effect of increasing the percentage of water and of the addition of an acid. Then, the SBs were enclosed in gel formulations to study the possibility of controlling the hydrolysis rate in the presence of a supramolecular encapsulating agent. 

Low molecular weight (LMW) gelators are small compounds able to form supramolecular gels thanks to non-covalent interactions like π-π stacking and hydrogen bonds [19,20,21,22,23]. Among them, peptide-based LMW gelators offer several advantages, such as easy synthesis, a high tunability of the gelator structure, which affects gel properties, and often the biocompatibility of the scaffold [24,25,26,27]. These molecules can respond to a variety of triggers (light, pH, ions, solvents, enzymes, ultrasounds) that cause the gelation process to start [28,29,30]. LMW gels can also be functionalized depending on the final application, with the introduction of fillers (graphene, nanoparticles, catalysts) [31,32,33,34], crystals [35,36], drugs or active ingredients [37,38,39]. 

In this work, we tested the hydrolysis of the SB in LMW supramolecular gels for the controlled release of perfumed compounds in comparison with the results obtained in solution. In particular, two gels were prepared using an EtOH/H_2_O mixture and two derivatives of L-Dopa, one possessing an ester moiety and the other the free carboxylic moiety. The effect of the acidic environment in the supramolecular media was also assessed, demonstrating the possibility to tune the fragrance release combining two means: the gel media and the pH of the medium.

The use of these supramolecular gels is a novelty in the study of the controlled release of fragrances. Peptide-based low molecular weight gelators are small molecules that are completely biocompatible and able to self-assemble into long fibers. The gels obtained by supramolecular self-assembly are often thixotropic, and can be adapted to any surface or used on the skin. The chemical properties (solvent, pH, presence of ions) of these gels may be easily tuned according to the reaction that should be catalyzed to release the fragrance. Moreover, we demonstrated that these gels offer the possibility of a high loading of profragrance (and thus of fragrance), as 1 mL of a gel contains 10 mg of gelator and 5 mg of Schiff base. The introduction of the four different Schiff bases in the gels does not affect their formation or their mechanical properties.

## 2. Results and Discussion

**Figure 1:**
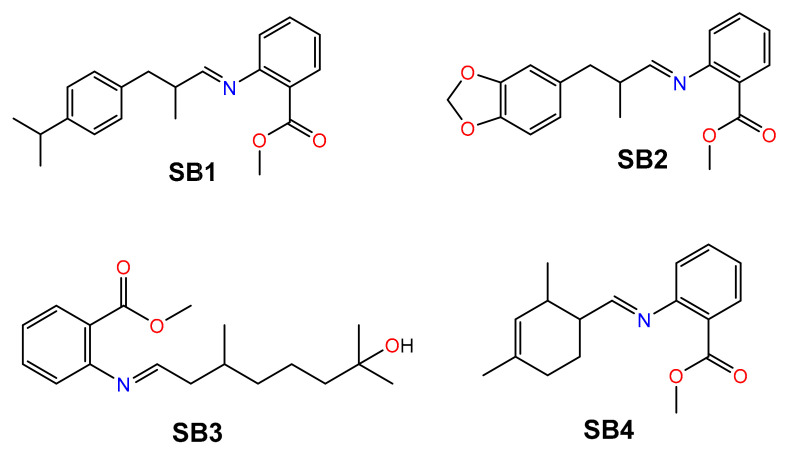
Structure of the four Schiff bases **SB1**–**SB4** discussed in this work.

Four SBs were synthesized, starting from methyl anthranilate (MA) and four odorant aldehydes (A): cyclamal (A1), helional (A2), hydroxycitronellal (A3) and triplal (A4), throughout the intermediate formation of a carbinolamine and the final release of a water molecule (Scheme S1) [16,40,41,42,43,44]. The aldehydes are all chiral compounds and are used in the racemic form. The structures of the final SBs (**SB1**–**SB4**) are reported in Figure 1. 

The synthesis of **SB1**–**SB4** was optimized after several attempts using different solvents and dehydrating agents (results not shown). In no cases were satisfactory yields obtained, so the condensation was repeated, eliminating the use of solvents and dehydrating agents by direct condensation of the neat reagents at 110 °C, as they are all liquids. 

We checked several ratios between the two reagents to obtain the best reaction conditions, determining the reaction conversion by HPLC-MS from the disappearance of methyl anthranilate, using a calibration curve (Figure S1, Tables S1 and S2). For the synthesis of **SB1**, **SB2** and **SB4**, the best results were obtained with an excess of the aldehyde with respect to methyl anthranilate in a 2:1 ratio. In contrast, the best yield for preparation of **SB3** was obtained with an excess of methyl anthranilate in a 2:1 ratio. The different behavior may be ascribed to the presence of a hydroxy group present in the chemical structure of A3 that increases its polarity and affects its reactivity. The optimal reaction conditions and the conversions for compounds **SB1**–**SB4** are summarized in Table 1. 

**Figure 2:**
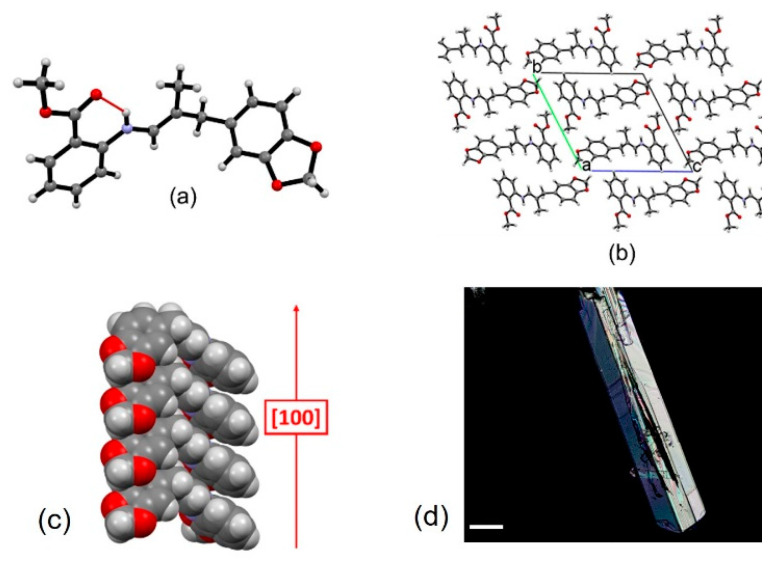
Structural features of crystalline **SB2**: (**a**) the asymmetric unit, showing the intramolecular S_1_^1^(6) hydrogen bonding interaction between the iminic nitrogen and carbonyl group, (**b**) crystal packing viewed down the a-axis, and (**c**) detail of the columnar stacking extending along the [100] crystallographic direction; (**d**) optical microscope image (polarized light) of the crystals of **SB2**. Scalebar is 100 μm.

The purification of the four SBs was frustrating. Any attempt to purify them by silica chromatography failed, as the imine group quickly hydrolyzed, producing a dramatic reduction of the final yields of SBs. Moreover, the purification by crystallization was successful only for **SB2**, which readily crystallized from methanol, so we could establish its structure by X-ray diffraction analysis (Figure 2 and Figure S2). The crystallization was favored by the presence of a racemic mixture.

Single-crystal XRD analysis for **SB2** confirms the molecular structure of the synthesized compound and reveals that it crystallizes in the triclinic P-1 space group with one molecule in the asymmetric unit (Figure 2; see Table S3 for details and Figure S3 for the Ortep plot). The only intermolecular interaction found is a strong intramolecular hydrogen bond [45,46] involving the iminic nitrogen and carbonyl group [N_N—H_∙∙∙O_C=O_ = 2.657 (2) Å] (see Figure 2), and according to the graph-set notation [47,48] it can be classified as type S_1_^1^(6). In contrast, no other intermolecular interactions are detected, and crystal cohesion is mainly due to dispersion forces, with **SB2** molecules stacking along the [100] crystallographic direction at a distance of ca 4.9 Å.

As the yield for the synthesis of the SB is always high, we studied the hydrolysis rate of the four unpurified SBs, **SB1**–**SB4**. We also studied the hydrolysis rate of the purified **SB2** (from now on **SB2P**) to check whether the presence of impurities affects the hydrolysis rate.

The stability of the compounds was assessed in EtOH/H_2_O solutions under three different conditions: 85:15 ratio, 70:30 ratio and 70:30 ratio with the addition of acetic acid (Table 2), as acidic conditions are known to favor the hydrolysis of Schiff bases. 

In ethanol/water mixtures, the hydrolysis is poor after 24 h except for **SB2P** and **SB3**. In contrast, with the addition of a small amount of acid, the hydrolysis results much faster for all SB (Table 2 and Table S4). The comparison between **SB2** and **SB2P** indicates that the starting pH of **SB2P** solutions is always lower than **SB2** solutions, as in this latter case some methyl anthranilate is still present in the mixture. Conversely, the hydrolysis of **SB2P** in acidic solution is slower than **SB2**, probably due to the slow reactivity of the microcrysals dispersed in the solution (Figure S5). 

To better control the hydrolysis rate of **SB1**–**SB4** and the fragrance release over time, which ideally should be completed within a few days, we inserted the four compounds inside two gelators derived from L-Dopa with different characteristics, as one is an ester (Boc-L-Dopa-(Bn)_2_-OMe, gelator (**A**) while the second displays an acidic moiety that should catalyze the SB hydrolysis (Boc-L-Dopa-(Bn)_2_-OH, gelator (**B**). We previously demonstrated that gelator **B** is very versatile, as it can form gels under several conditions in low concentrations [35]. 

The ability of **A** and **B** to form strong and self-supporting gels was studied using the same solvents ratio selected in Table 2 to check whether these gelators are suitable for this study. Gelator **A** mimics the condition obtained with the solvent mixtures, so its gelation ability was studied in 85:15 and 70:30 solvent ratio, while gelator **B** mimics the condition obtained with the solvent mixture with the addition of acetic acid, so it was studied only in 70:30 solvent ratio. To prepare the gels, we used the solvent switch method, dissolving the gelator in EtOH and then adding water to trigger gel formation (for more details see Section 3). 

The formation of strong and self-supporting gels occurred in all conditions (Figure S6). The analysis of the samples was pursued with the study of the mechanical properties of the gels by means of a rheometer. New gel samples were prepared in 2 mL Sterilin Cups^®^ to check their stiffness. The final pH values were also measured and were very similar to the pH values of the solutions. The results are shown in Table 3, while the complete series of the amplitude sweep measurements are reported in Figure S7.

**Figure 3:**
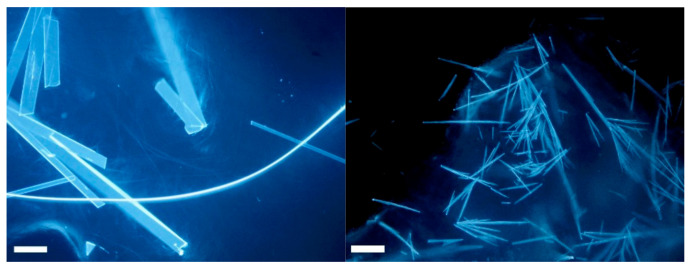
(**left**) Microscope image in epifluorescence mode of a piece of gel **A** containing **SB2P**; (**right**) microscope image in epifluorescence mode of a piece of gel **B** made with **SB2P**. Scalebar is 100 µm.

As gels were obtained in all conditions, we prepared ten gels containing the selected gelator in 1% *w*/*v* concentration and the selected SB in 0.5% *w*/*v* concentration (see Materials and Methods for details). The inspection with a microscope of the gels containing **SB2P** highlights that some crystals are contained in the gels, as we previously observed for the solutions (Figure 3).

**Figure 4:**
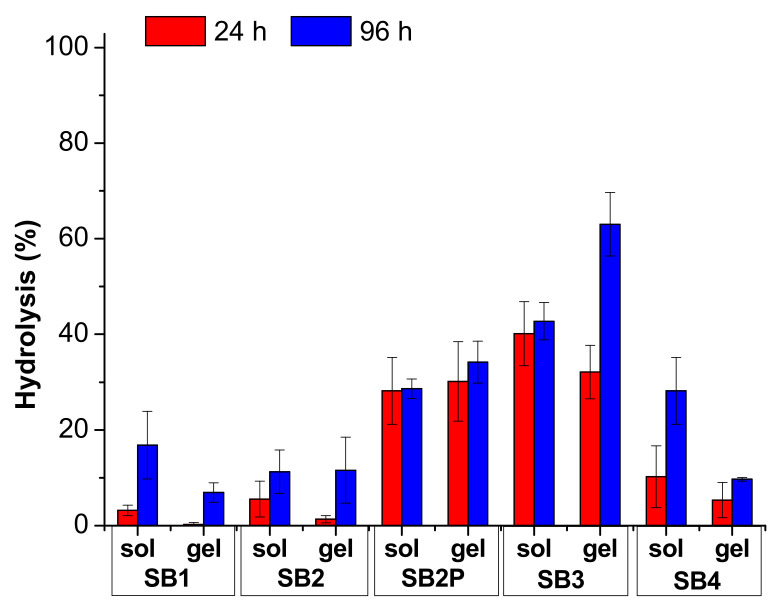
Hydrolysis results for the SB under neutral conditions. The data are reported as mean value and standard deviation.

**Figure 5:**
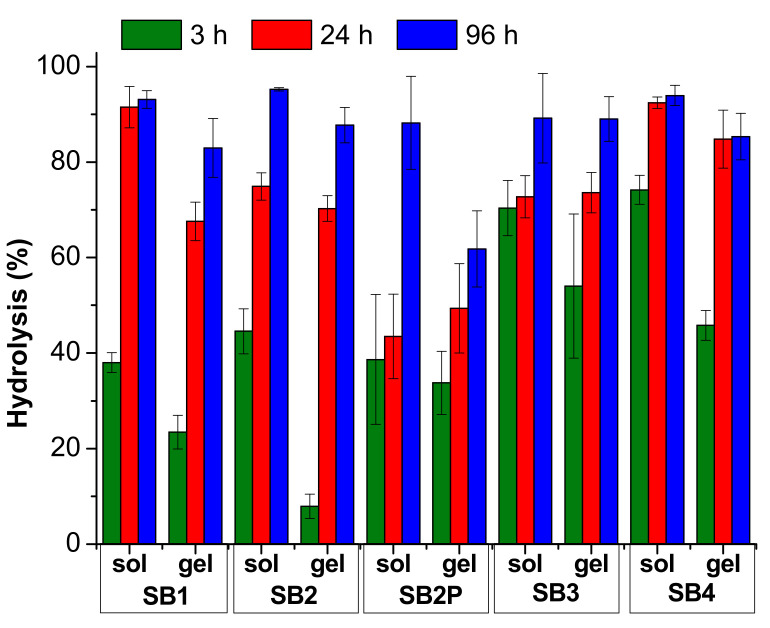
Hydrolysis results for the SB under acidic conditions. The data are reported as mean value and standard deviation.

The hydrolysis results are reported in Figure 4 and Figure 5 and in Tables S5 and S6. In Figure 4 and Table S5, we compared the results obtained under neutral conditions, both in solution and in gel. For these measures we analyzed the medium in 70:30 solvent ratio only. In Figure 5 and in Table S6, we collected the results for the hydrolysis under acidic conditions, both in solution and in gel.

The comparison between the results in solution and in gel indicates that the acidic catalysis is always crucial for the hydrolysis reaction, as could be foreseen. Indeed, the gel prepared with gelator A with a pH ≈ 6 is not able to significatively enhance the hydrolysis rate in any condition, as only **SB3** reaches 63% hydrolysis after 96 h. This medium is strongly preferred if a gel is required with a very slow odor release, lasting weeks, but with reduced intensity. 

In contrast, the gel prepared with gelator B has a final pH < 4 due to the acidic moiety contained in the chemical structure that catalyzes the hydrolysis. It is worth noting that, in any case, the hydrolysis in solution is much faster than in gel, although the pH is very similar. This medium should be preferred if the odor release is required within few days.

## 3. Materials and Methods

### 3.1. Synthesis and Characterization of ***SB1***–***SB4***

Solvents were dried by distillation before use. All reactions were carried out in dried glassware. The melting point of the compound was determined in open capillaries and is uncorrected. High-quality infrared spectra (64 scans) were obtained at 2 cm^−1^ resolution with an FT-IR Bruker/Billerica/US/MA Alpha System spectrometer. NMR spectra were recorded with a Varian/Palo Alto/US/CA Inova 400 spectrometer at 400 MHz (^1^H NMR), at 100 MHz (^13^C NMR). Chemical shifts are reported in δ values relative to the solvent peak. HPLC-MS was used to check the purity of compounds. HPLC-MS analysis was carried out with an Agilent 1260 Infinity II liquid chromatograph coupled to an electrospray ionization mass spectrometer (LC-ESI-MS), using a Phenomenex Gemini C18 −3μ—110 Å column, and H_2_O/CH_3_CN with 0.2% formic acid as acid solvent at 40 °C (positive ion mode, m/z = 50–2000, fragmentor 70 V).

### 3.2. Hydrolysis Study in Solution

After the reaction has undergone the optimum time for the specific Schiff base synthesis, the product was dissolved in 8.5 mL of ethanol and 1.5 mL of water in a 10 mL volumetric flask. HPLC-MS analysis was carried out at the desired time intervals to analyze the hydrolysis of the Schiff base, by taking a withdrawal of 100 μL of the SB solution and adding 900 μL of ACN. The same procedure was followed for the hydrolysis in the 70:30 EtOH/H_2_O solution (solution A), but the product was dissolved in 7.0 mL of ethanol and 3.0 mL of water in a 10 mL volumetric flask. 

### 3.3. Hydrolysis Study in Acidic Solution

After the synthesis, each Schiff base was dissolved in EtOH to obtain a solution with a known concentration. The volume of this solution required to add 0.5% *w*/*v* concentration of SB (5 mg/mL) was calculated. The required volume of EtOH (700 μL—volume of EtOH/SB solution) was added, together with 300 μL of Milli-Q^®^ H_2_O. The mmol of gelator were replaced with mmol of glacial acetic acid to obtain a similar pH and added to the solution. To take the hydrolysis value at different times, different vials were prepared, all containing the same SB/acetic acid ratio.

### 3.4. Gel Preparation

After the synthesis, each SB was dissolved in EtOH to obtain a solution with a known concentration. The volume of this solution required to add 0.5% *w*/*v* concentration of SB (5 mg/mL) in the gel was calculated. 10 mg (1% *w*/*v* concentration) of gelator (A or B) was weighed into a vial and dissolved in the required volume of EtOH (700 μL—volume of EtOH/SB solution). The SB/EtOH solution was also added to the gelator. The solution was sonicated until the dissolution of the two compounds was achieved. Finally, to trigger the formation of the gel, 300 μL of Milli-Q^®^ H_2_O was added to the vial and immediately gently shaken to achieve a homogeneous gel. To take the hydrolysis value at different times, it was necessary to prepare several vials containing the same gel, because to improve the homogenization of the sample tested with the HPLC, the whole gel had to be dissolved in acetonitrile. 

### 3.5. Optical Microscope Images

The optical microscope images were recorded using a Nikon (Minato, Japan) 13 ECLIPSE Ti2 Inverted Research Microscope with a 10× magnifier. The images of the crystals were taken using polarized light. The images of the gels were instead taken in epifluorescence mode, using a fluorescent filter cube V-2A and an excitation LED (λ = 395 nm). The gel samples were analyzed while wet. 

### 3.6. Single-Crystal X-ray Diffraction

Single-crystal data for compound **SB2** were collected at RT on an Oxford XCalibur S CCD diffractometer equipped with a graphite monochromator (Mo-K_α_ radiation, λ = 0.71073 Å). The structure was solved by the intrinsic phasing method with SHELXT [49] and refined on F^2^ by full-matrix least squares refinement with SHELXL [50] implemented in the Olex2 software [51]. All non-hydrogen atoms were refined anisotropically applying the rigid-body RIGU restraint [52]. H_CH_ atoms for all compounds were added in calculated positions and refined riding on their respective carbon atoms. Data collection and refinement details are listed in Table S3. The Mercury [53] program was used to calculate intermolecular interactions and for molecular graphics. Crystal data can be obtained free of charge via www.ccdc.cam.ac.uk/conts/retrieving.html (accessed on 9 March 2022), (or from the Cambridge Crystallographic Data Centre, e-mail: deposit@ccdc.cam.ac.uk); CCDC number 2149692. 

### 3.7. Rheology

The rheological measurements were performed using an Anton Paar (Graz, Austria) MCR102 rheometer. The gels were directly prepared in the Thermo Fisher Scientific (Waltham, MA, USA) Sterilin cup, which fits in the rheometer. A vane and cup measuring system was used, setting a gap of 2.1 mm. Oscillatory amplitude sweep experiments (γ: 0.01–100%) were performed in triplicate at 23 °C using a constant angular frequency of 10 rad/s, 16 h after the addition of water, to allow a complete gel formation.

## 4. Conclusions

With the aim of preparing materials able to release odorant molecules over several days, we studied the hydrolysis of four SB profragrances in different media to tune their hydrolysis time as a function of the medium acidity and the consequent release of odorant aldheydes and methyl anthranilate. In water/ethanol solutions, the hydrolysis is very slow, while it is very fast when an acid is added, as the hydrolysis is complete within 24 h. Neither of those two conditions is desirable for a release lasting a few days. So, the SB profragrances were trapped in two gel media, both obtained with low molecular weight gelators in 1% concentration in a ethanol/water mixture. Under these conditions, the SB hydrolysis rate is modified, as it is enhanced in the neutral gel (pH ≈ 6) compared to the neutral solution, and slowed in the acidic gel (pH < 4) compared to the acidic solution. Both media release the odor molecules within some days and may be chosen as a function of the required application. These results prove the importance of the gel medium to control the hydrolysis releasing the fragrance over the time, and prove that the possibility to tune the pH or other chemical properties of these gels is an important feature in the design of the controlled release.

## Figures and Tables

**Table 1:** Reaction conditions chosen for the synthesis of **SB1**–**SB4**.

Schiff Base	Aldehyde	Ratio A:MA	Time (min)	Conversion (%)
**SB1**	A1	2:1	30	93.3
**SB2**	A2	2:1	30	97.8
**SB3**	A3	1:2	120	82.0
**SB4**	A4	2:1	60	81.8

**Table 2:** Hydrolysis of SB after 24 h in ethanol/water mixtures reported as mean value and standard deviation.

Schiff Base	EtOH/H_2_O Ratio	Acetic Acid (mmol/mL)	Starting pH	Hydrolysis at 24 h (%)
**SB1**	85:15	-	5.92	3.23 ± 1.87
**SB1**	70:30	-	5.03	3.20 ± 1.10
**SB1**	70:30	0.02	3.55	91.49 ± 4.34
**SB2**	85:15	-	5.71	2.58 ± 0.51
**SB2**	70:30	-	5.14	5.55 ± 3.76
**SB2**	70:30	0.02	3.62	74.91 ± 2.86
**SB2P**	85:15	-	4.81	23.08 ± 7.56
**SB2P**	70:30	-	4.83	28.18 ± 7.00
**SB2P**	70:30	0.02	3.60	43.49 ± 8.83
**SB3**	85:15	-	5.66	43.06 ± 0.60
**SB3**	70:30	-	5.27	40.14 ± 6.72
**SB3**	70:30	0.02	3.57	72.73 ± 4.41
**SB4**	85:15	-	5.93	11.93 ± 1.27
**SB4**	70:30	-	5.69	10.23 ± 6.47
**SB4**	70:30	0.02	3.74	92.41 ± 1.19

**Table 3:** Summary of the gel properties (G’ and G’’ were taken from amplitude sweep at shear strain 0.03%).

Gelator	Ratio EtOH/H_2_O	G’ (kPa)	G” (kPa)	Final pH
A (ester moiety)	85:15	125.41 ± 23.02	41.41 ± 3.89	6.42 ± 0.16
A (ester moiety)	70:30	87.72 ± 22.46	16.49 ± 3.21	5.98 ± 0.19
B (acidic moiety)	70:30	43.01 ± 11.02	12.04 ± 3.10	3.80 ± 0.03

## Data Availability

Crystal data can be obtained free of charge via www.ccdc.cam.ac.uk/conts/retrieving.html (accessed on 9 March 2022), (or from the Cambridge Crystallographic Data Centre, 12 Union Road, Cambridge CB21EZ, UK; fax: +44-1223-336-033; or e-mail: deposit@ccdc.cam.ac.uk); CCDC number 2149692.

## Supplementary Materials

The following supporting information can be downloaded at: https://www.mdpi.com/article/10.3390/ijms23063105/s1.
