# Supplementary material for: Controlled Hydrolysis of Odorants Schiff Bases in Low-Molecular-Weight Gels
Source: Int J Mol Sci. 2022 Mar 13;23(6):3105. doi: 10.3390/ijms23063105 (PMC8952255; doi:10.3390/ijms23063105)
Supplement: Supplementary file 1 [file ijms-23-03105-s001.zip › ijms-1617891-supplementary.pdf]

Supporting Information

Controlled hydrolysis of odorants Schiff bases in low-molecular-weight gels

Gloria Nicastro [1], Louise Mary Black [2], Paolo Ravarino [1], Simone d'Agostino [1], Davide Faccio [1], Claudia Tomasini [1,*], and Demetra Giuri [1,*]

1 Dipartimento di Chimica Giacomo Ciamician, Università di Bologna, Via Selmi, 2, 40126 Bologna, Italy; gloria.nicastro@studio.unibo.it (G.N.); louise.black@studio.unibo.it (L.M.B.;) paolo.ravarino2@unibo.it (P.R.); simone.dagostino2@unibo.it (S.D.A.); davide.faccio2@unibo.it (D.F.)
2 School of Chemistry, University of Glasgow, Glasgow G12 8QQ, UK; louise.black@studio.unibo.it
* Correspondence: demetra.giuri2@unibo.it; claudia.tomasini@unibo.it

**Scheme S1.** Synthesis of compound **SB1-SB4**	S2
Preparation of compounds **SB1-SB4**	S3
MS spectra of **SB1-SB4**	S4-S7
^{1}H NMR, ^{13}C NMR and IR spectra of **SB2P**	S8-S9
**Figure S1.** Methyl anthranilate calibration curve	S11
Schiff base synthesis: A:MA ratio screening	S12
**Table S1.** Screening of the optimal A:MA ratio for each SB synthesis	S13
Schiff base synthesis: time screening	S14-S15
**Table S2.** Screening of the optimal time for each SB synthesis	S16
**Figure S2.** Optical microscope images of the crystals of SB2P	S17
**Table S3.** Crystal data and refinement details for crystalline SB2P	S18
**Figure S3.** Ortep drawing of **SB2P**	S19
**Figure S4.** Calibration curve Helional Schiff base (crystallised)	S20
Hydrolysis in solution: analysis method	S21
**Table S4.** pH values for solutions and gels for all the SB	S22
**Figure S5.** Photograph of SB2P solutions after sonication	S23
**Figure S6.** Photograph of the gels obtained with gelators A and B	S23
**Figure S7.** Amplitude sweep measurements for the gels prepared	S24
**Table S5.** Hydrolysis results for the SB under neutral conditions.	S25
**Table S6.** Hydrolysis results for the SB under acidic conditions.	S25

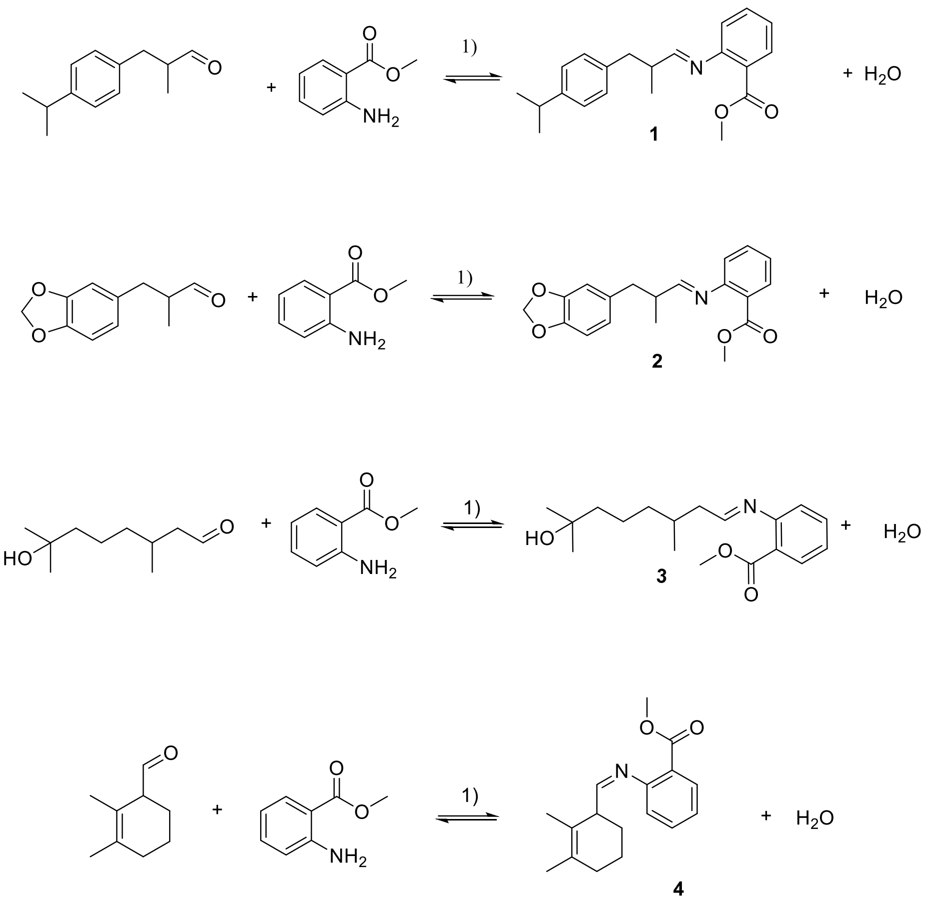

Scheme S1. Synthesis of compound **SB1-SB4**. Reagents and conditions: 1) T = 110 °C.

Preparation of compounds SB1-SB4.

SB1. Cyclamal aldehyde (126 mg, 0.662 mmol) and methyl anthranilate (50 mg, 0.331 mmol) were mixed in 2:1 ratio in a vial and placed in an oil bath preheated at 110 °C. The reaction mixture was stirred for 30 min. Then the crude was dissolved in EtOH and the conversion was quantified using an HPLC-MS. HPLC-MS (API-ES): 17.87 min, $[M+H]^+$=324.

SB2. Helional aldehyde (127 mg, 0.662 mmol) and methyl anthranilate (50 mg, 0.331 mmol) were mixed in 2:1 ratio in a vial and placed in an oil bath preheated at 110 °C. The reaction mixture was stirred for 30 min. Then the crude (**SB2**) was dissolved in EtOH and the conversion was quantified using an HPLC-MS. The reaction was also repeated on 2.60 mmol of aldehyde (1.30 mmol of MA) and the crude was dissolved in methanol (10 mL), heated until completely solubilization and left to crystallise overnight. The crystals (**SB2P**) were then filtered and washed several times with cold methanol (yield: 22.4%).

SB2P. HPLC-MS (API-ES): 11.68 min, $[M+H]^+$=326. M.p. = 82-84°C; IR-ATR: ν = 3302, 3261, 1658, 1606, 1584, 1520, 1483, 1435 cm^{-1}; ^1H NMR ($CDCl_3$, 400MHz) δ: 1.67 (3H, d, J=1.6 Hz, C-CH_3), 3.28 (2H, bs, Ar-CH_2), 3.87 (3H, s, O-CH_3), 5,91 (2H, s, O-CH_2-O), 6.38 (2H, dd, J=1.2, 10.4 Hz, Ar-CH), 6.69 (4H, m, Ar-CH), 6,91 (1H, dd, J=1.2, 8.8 Hz, Ar-CH), 7.36 (1H, m, Ar-CH), 7.91 (1H, dd, J=1.6, 8.0 Hz, Ar-CH), 9.51 (1H, J=10.4 Hz, CH=N); ^{13} C-NMR ($CDCl_3$) δ: 14.50, 42.80, 51.68, 100.75, 107.98, 109.08, 110.23, 111.66, 115.42, 116.08, 121.14, 121.53, 131.66, 134.20, 134.63, 145.81, 146.75, 147.58, 169.05.

SB3. Hydroxy citronellal aldehyde (57 mg, 0.331 mmol) and methyl anthranilate (100 mg, 0.662 mmol) were mixed in 1:2 ratio in a vial and placed in an oil bath preheated at 110 °C. The reaction mixture was stirred for 120 min. Then the crude was dissolved in EtOH and the conversion was quantified using an HPLC-MS. HPLC-MS (API-ES): 10.43 min, $[M+H]^+$=306.

SB4. Triplal aldehyde (91.5 mg, 0.662 mmol) and methyl anthranilate (50 mg, 0.331 mmol) were mixed in 2:1 ratio in a vial and placed in an oil bath preheated at 110 °C. The reaction mixture was stirred for 60 min. Then the crude was dissolved in EtOH and the conversion was quantified using an HPLC-MS. HPLC-MS (API-ES): 14.65 min, $[M+H]^+$=272.

MS of compound **SB1**.

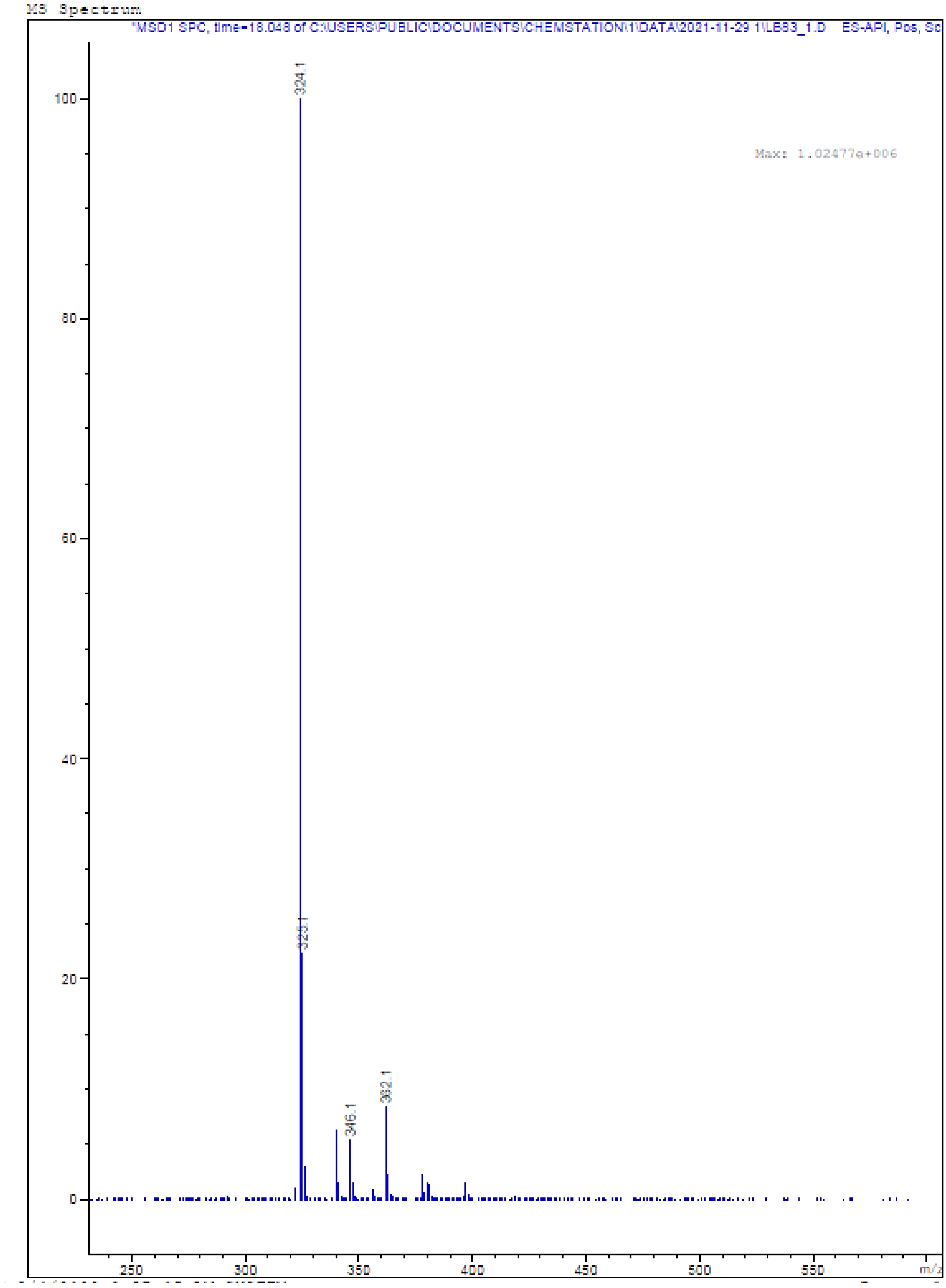

MS of **SB2**

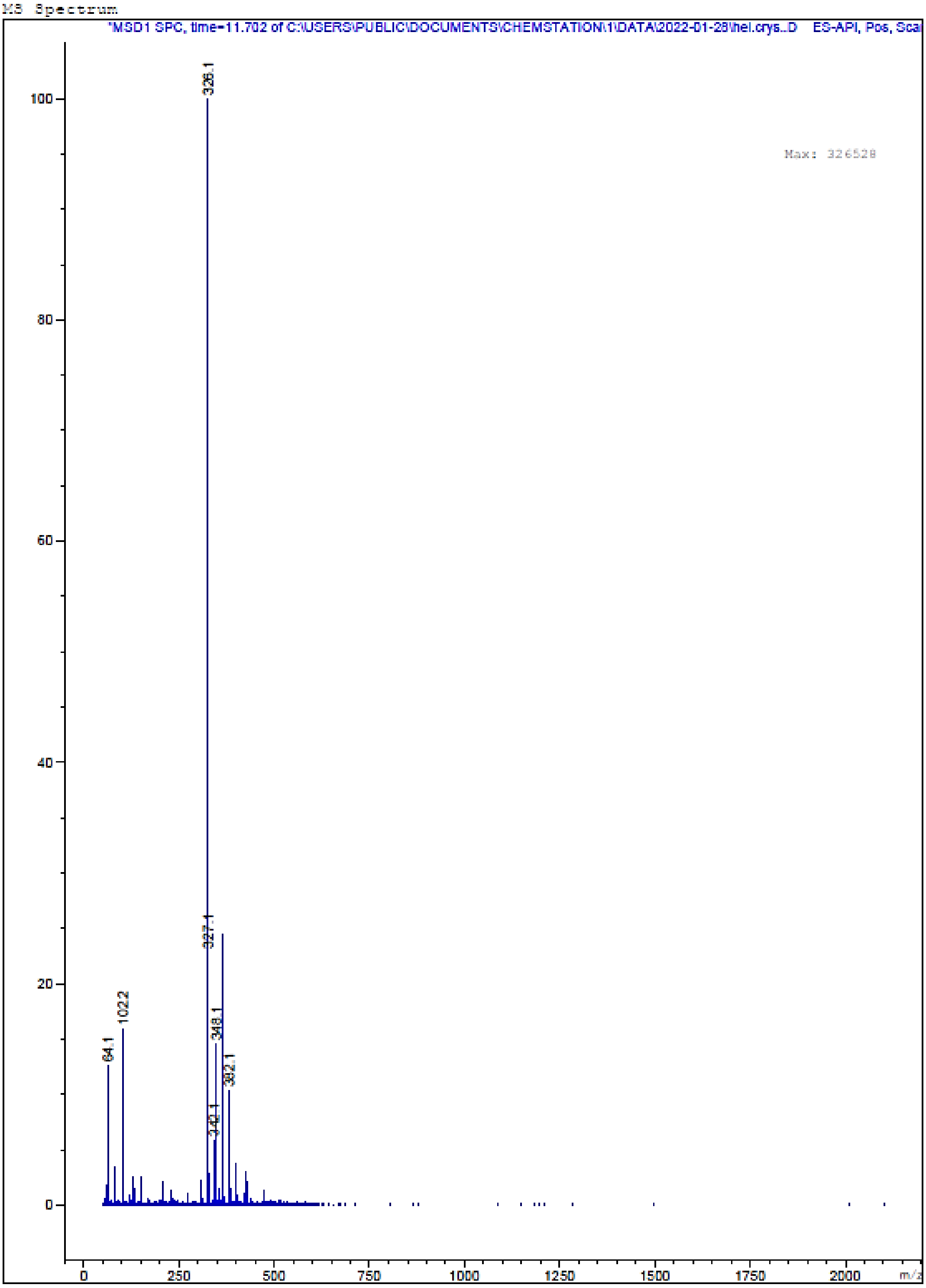
MS Spectrum
*MSD1 SPC, time=11.702 of C:\USERS\PUBLIC\DOCUMENTS\CHEMSTATION\1\DATA\2022-01-28\hei.crys..D ES-API, Pos, Sca
Max: 326528
326.1
327.1
102.2
64.1
348.1
382.1
342.1
m/z

MS of compound **SB3**

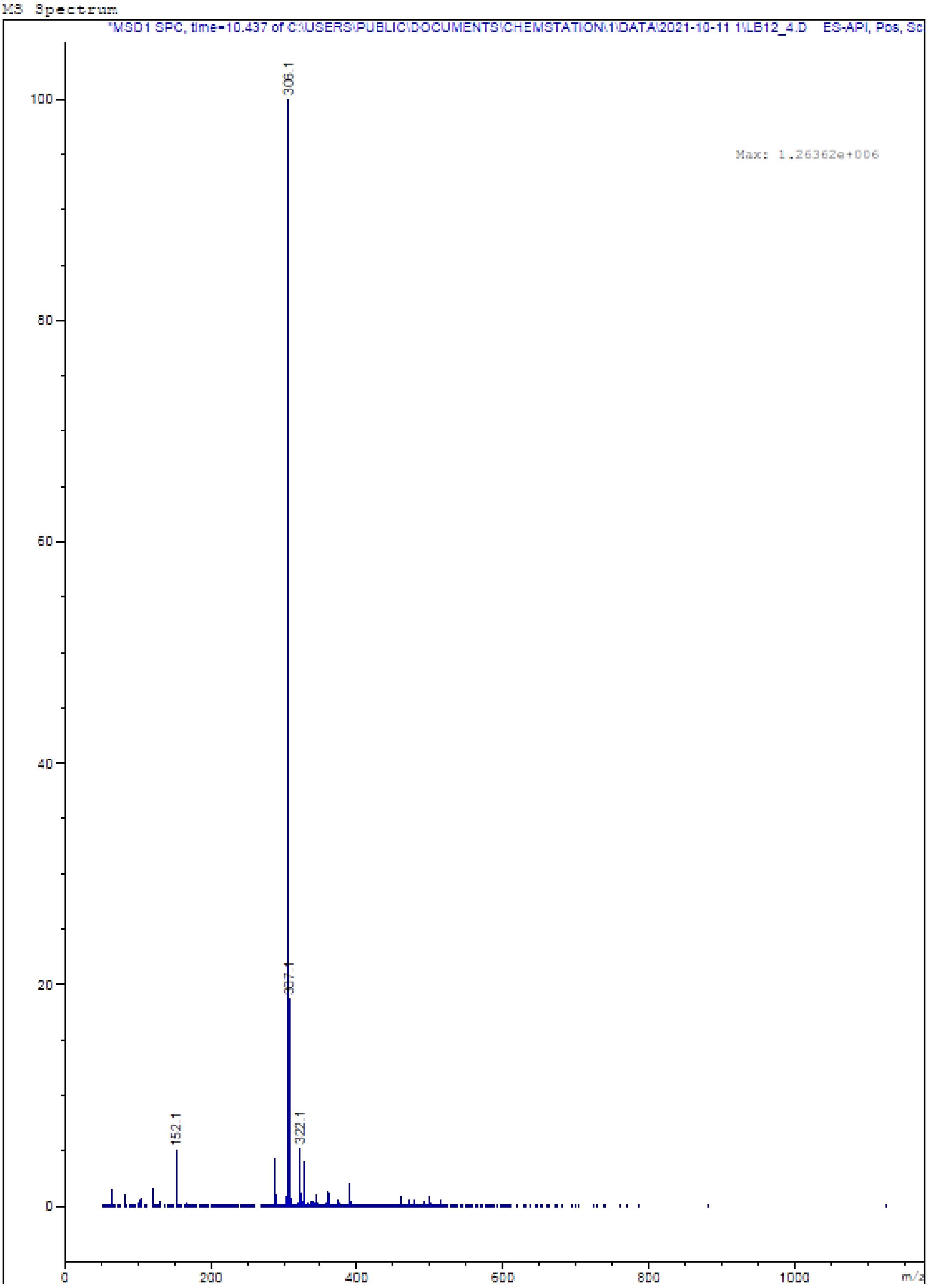

MS of compound **SB4**

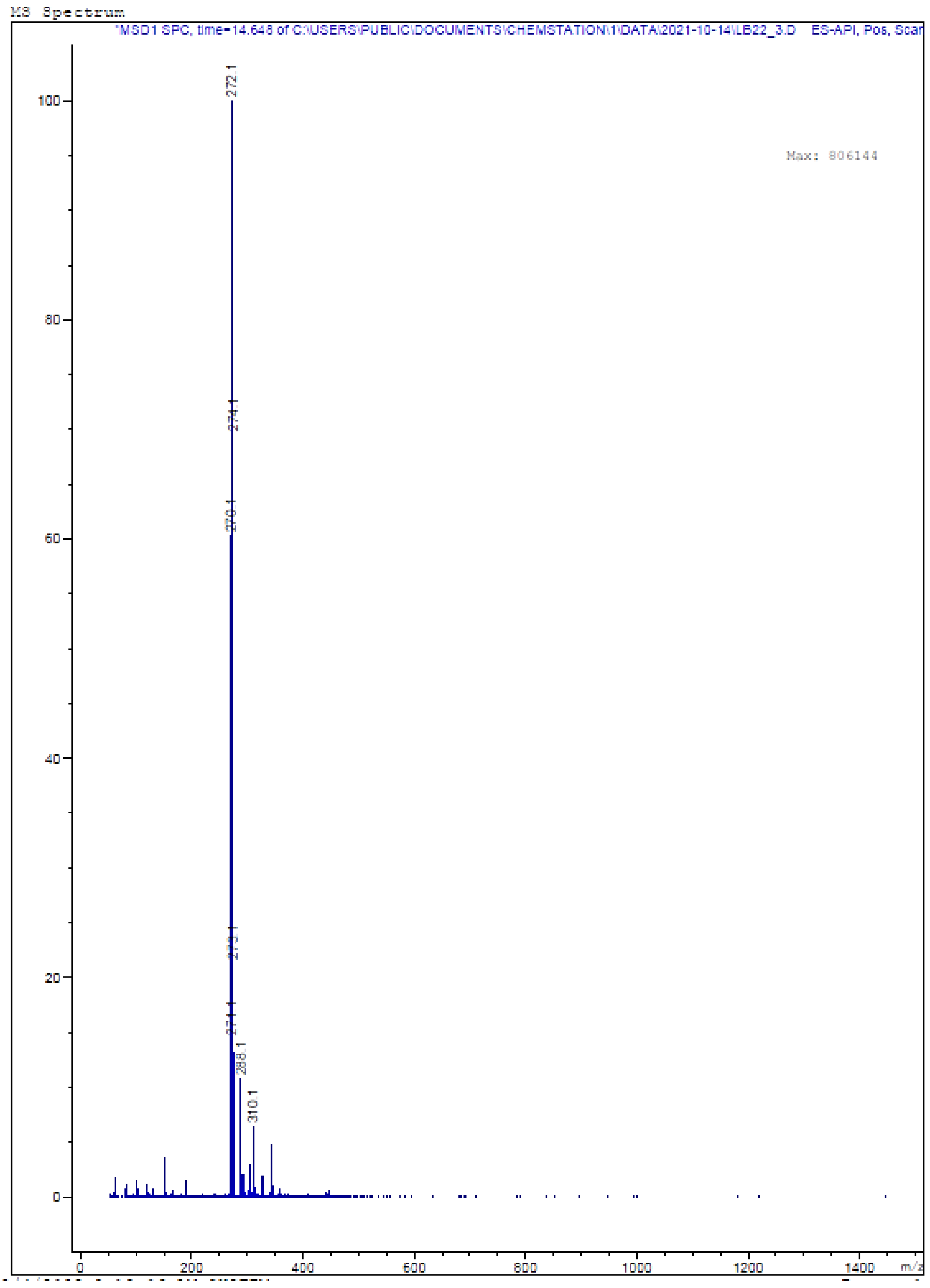

^{1}H-NMR spectrum of compound **SB2P**

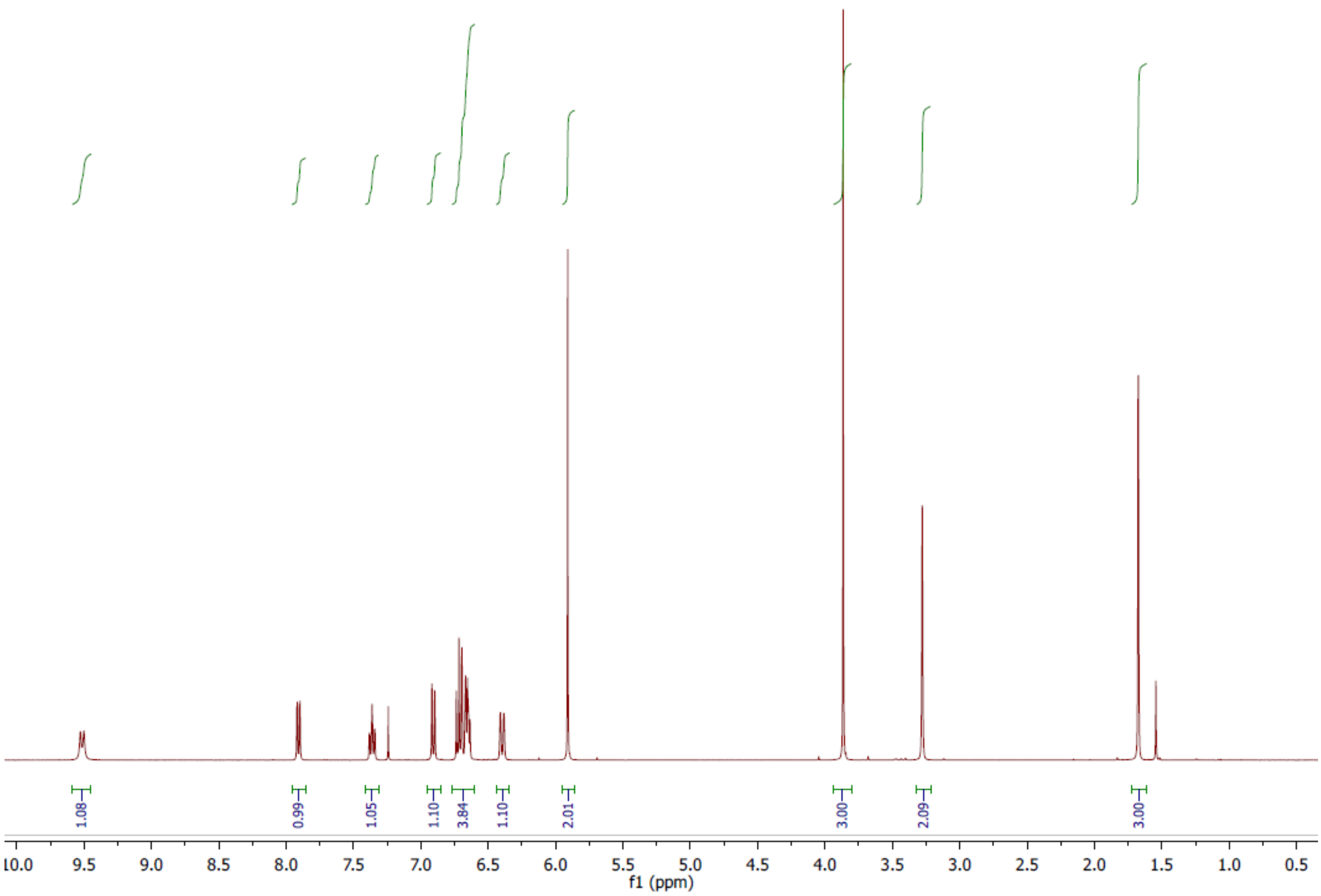

^{13}C NMR spectrum of compound **SB2P**

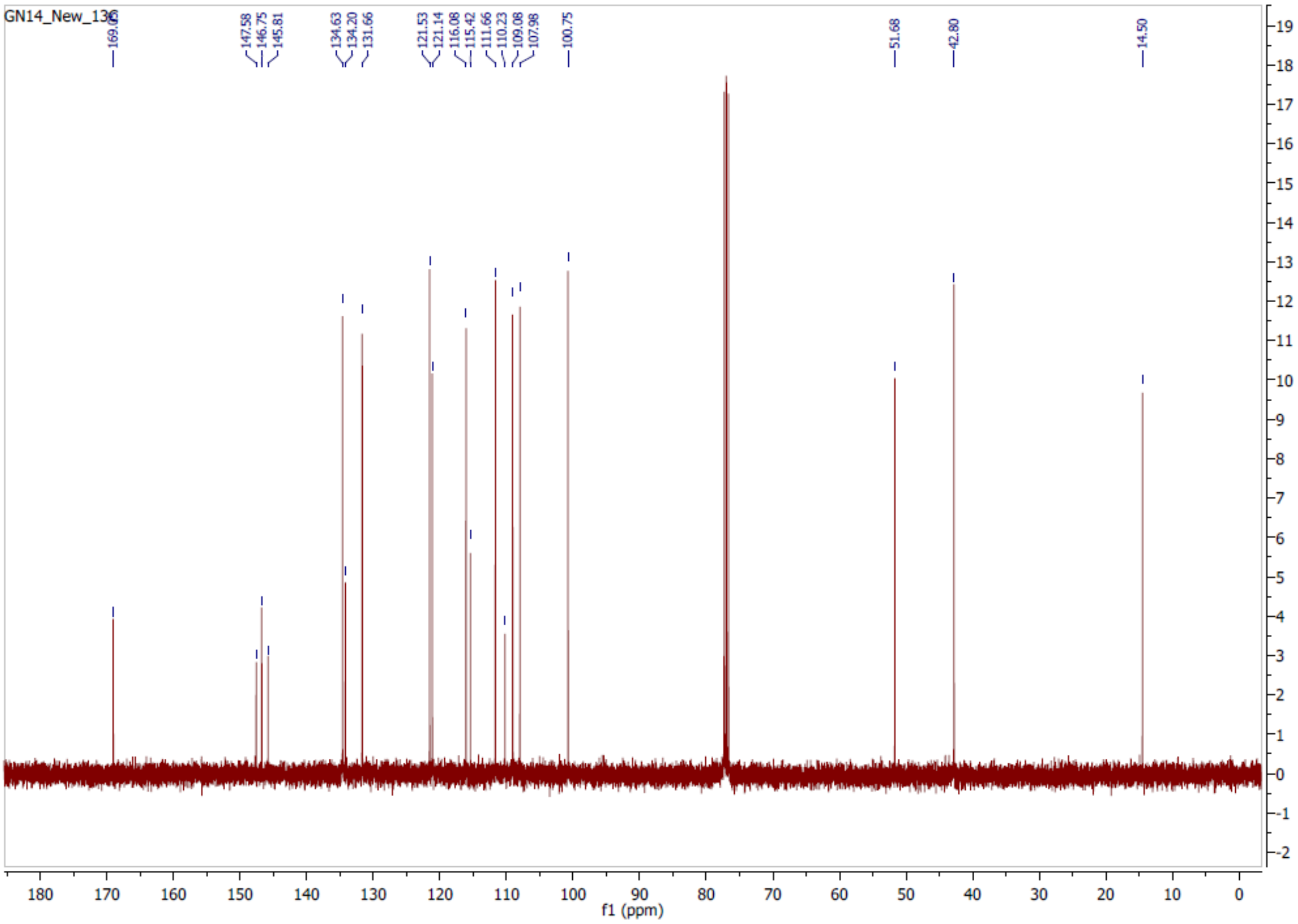

IR spectrum of compound **SB2P**

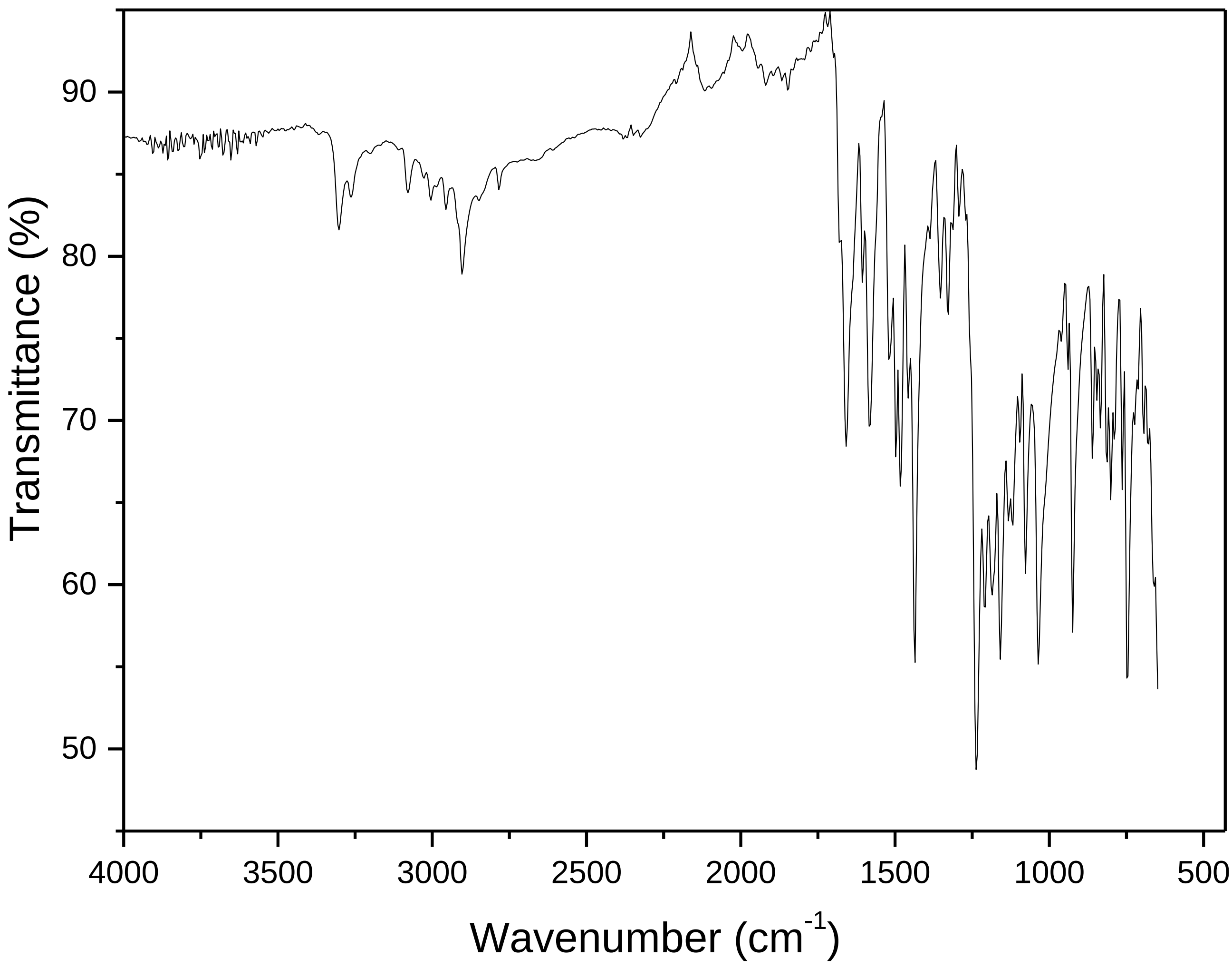

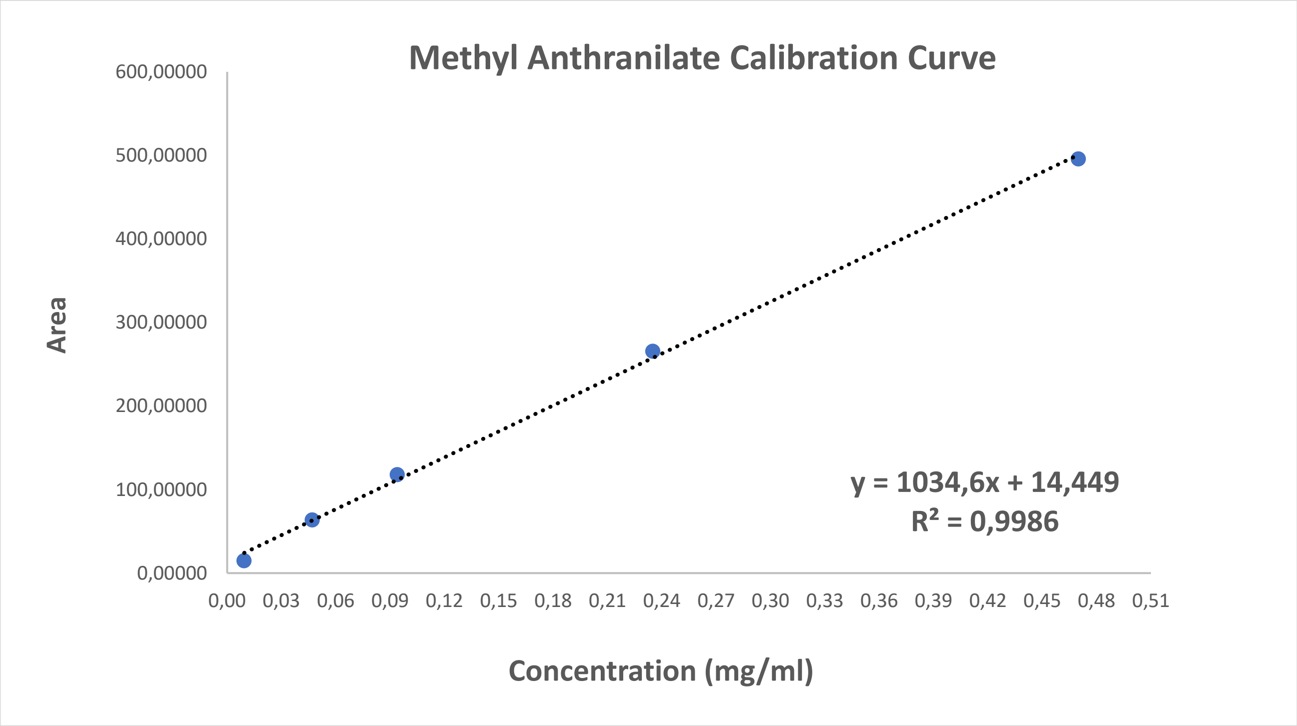

Figure S1. Methyl anthranilate calibration curve.

The calibration curve was built starting from a solution of methyl anthranilate (9.4 mg) in acetonitrile (10 mL). All the dilutions of this mother solution were injected in a HPLC-MS (DAD = 280 nm).

**Concentration (mg/ml)**	**Area**
0,0094	14,64658
0,0470	63,64524
0,0940	117,77985
0,2350	265,48605
0,4700	495,65579

Schiff base synthesis: A:MA ratio screening

The Schiff bases (SB) were prepared by mixing methyl anthranilate (MA) and aldehyde (A) at different equivalence ratio A:MA. Then, the mixture was placed in a preheated oil bath at 110 °C and continuously stirred using the magnetic stirrer. After 1 h, the entire contents of the vial were dissolved in a 5 ml standard flask using ethanol. This was diluted by dissolving 100 µl of this solution into a 3 ml standard flask using ACN. A HPLC-MS sample was taken from the ACN solution.

Method 1.

The concentration of unreacted MA after 1 h is determined from the calibration curve and, taking into account the dilution factor, the mg of unreacted MA were calculated. Assuming that all the MA consumed is converted into SB, the quantity of unreacted MA was subtracted from the initial mass of MA, obtaining the mass of reacted MA and the conversion as follows.

$$hx_0 = quantity\ of\ MA\ at\ the\ beginning\ of\ the\ reaction\ (in\ mmol)$$

$$x_1 = quantity\ of\ MA\ at\ the\ end\ of\ the\ reaction\ (in\ mmol)$$

$$x = quantity\ of\ SB\ at\ the\ end\ of\ the\ reaction\ (in\ mmol)$$

$$x = hx_0 - x_1$$

The constant h has known values and according to the ratio between MA and A it can be:

- 1, if the MA is the limiting reagent
- 2, if the quantity of MA used is the double of the limiting reagent

$$Conversion\ (\%) = \frac{\boldsymbol{hx_0 - x_1}}{\boldsymbol{x_0}} \times \mathbf{100}$$

Table S1. Screening of the optimal A:MA ratio for each SB synthesis

Schiff base	Aldehyde	Ratio A:MA	Time (min)	Conversion (%)
**1**	**A1**	**2:1**	**60**	**93.28**
1	A1	1:1	60	63.77
1	A1	1:2	60	77.31
**2**	**A2**	**2:1**	**60**	**95.13**
2	A2	1:1	60	82.38
2	A2	1:2	60	70.23
3	A3	2:1	60	17.56
3	A3	1:1	60	21.61
**3**	**A3**	**1:2**	**60**	**79.46**
**4**	**A4**	**2:1**	**60**	**81.73**
4	A4	1:1	60	44.72
4	A4	1:2	60	75.98

Schiff base synthesis: time screening

To study the optimal time for each Schiff base synthesis, MA and A were mixed at the correct equivalence ratio. Then, placed in a preheated oil bath at 110 °C ± 5 and continuously stirred using the magnetic stirrer. A HPLC-MS sample was taken and weighed at the various time intervals, and then diluted into 1 ml of acetonitrile (ACN). A methyl anthranilate calibration curve was constructed to allow the determination of its concentration (mg/ml) left after the reaction. The area of the methyl anthranilate peak from the 280 nm signal was used in the calibration curve equation.

Method 2.

Taking **N** mg of mixture at the first withdrawal t_1 it is possible to know the amount of MA left in the mixture from the calibration curve.

If the same amount **N** was withdrawn at the time t_0, it would have been composed as follow.

Case 1: the ratio A:MA is 1:2

$$X_A + Y_{MA} = N$$

Where X_A and Y_{MA} are the masses of aldehyde and methyl anthranilate respectively and can be expressed as product of the quantity of matter and the molecular weight of each one.

$$x_0 MW_A + 2x_0 MW_{MA} = N$$

Where x_0 is the quantity of matter of the limiting reagent inside the mixture at time t_0 expressed in mmol. Therefore:

$$\boldsymbol{x_0 = \frac{N}{MW_A + 2MW_{MA}}}$$

The time at which the withdrawal was taken is called t_1. The amount of SB produced at time t_1 is called x and can be obtained from the calibration curve of MA, assuming that all the MA consumed is converted into SB. The amount of MA left at time t_1 is called x_1, therefore:

$$\boldsymbol{x = 2x_0 - x_1}$$

$$\boldsymbol{Conversion}\ (\%) = \frac{\boldsymbol{2x_0 - x_1}}{\boldsymbol{x_0}} \times \mathbf{100}$$

Case 2: the ratio A:MA is 2:1

$$2x_0 MW_A + x_0 MW_{MA} = N$$

And therefore:

$$x_0 = \frac{N}{2MW_A + MW_{MA}}$$

quantity of SB $x = x_0 - x_1$

$$\boldsymbol{Conversion}\ (\%) = \frac{\boldsymbol{x_0 - x_1}}{\boldsymbol{x_0}} \times \mathbf{100}$$

Case 3: the ratio A:MA is 1:1

$$x_0 MW_A + x_0 MW_{MA} = N$$

And therefore:

$$x_0 = \frac{N}{MW_A + MW_{MA}}$$

quantity of SB $x = x_0 - x_1$

$$\boldsymbol{Conversion}\ (\%) = \frac{\boldsymbol{x_0 - x_1}}{\boldsymbol{x_0}} \times \mathbf{100}$$

Table S2. Screening of the optimal time for each SB synthesis, using the A:MA ratio selected.

Schiff base	Aldehyde	Ratio A:MA	Time (min)	Conversion (%)
**1**	**A1**	**2:1**	**30**	**93.28**
1	A1	2:1	60	79.89
1	A1	2:1	120	82.50
**2**	**A2**	**2:1**	**30**	**97.76**
2	A2	2:1	60	95.13
2	A2	2:1	120	97.17
3	A3	1:2	30	45.55
3	A3	1:2	60	79.46
**3**	**A3**	**1:2**	**120**	**82.04**
4	A4	2:1	30	71.62
**4**	**A4**	**2:1**	**60**	**81.73**
4	A4	2:1	120	81.97

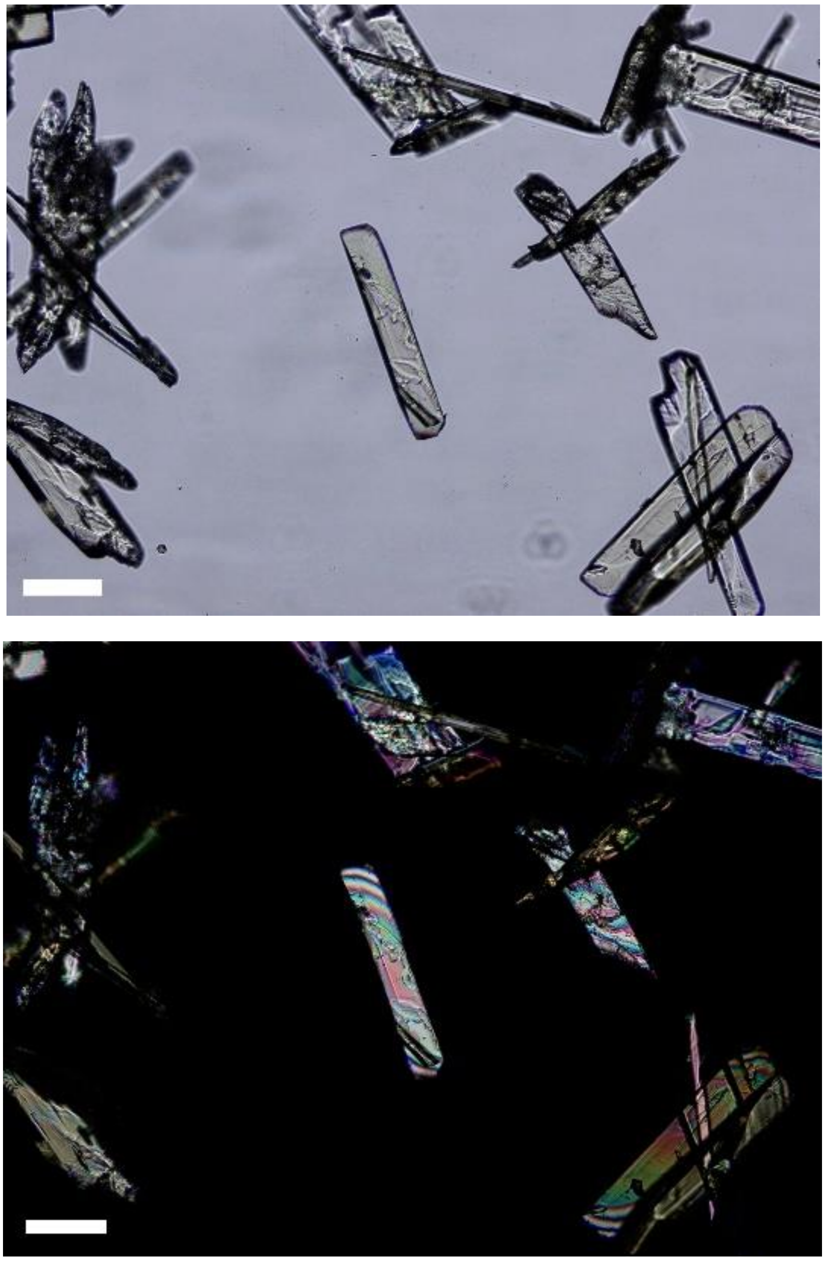

Figure S2. Optical microscope images of the crystals of **SB2P** (polarized light on the bottom). Scalebar is 100 μm.

Table S3. Crystal data and refinement details for crystalline **SB2**.

	**SB2**
**Formula**	$C_{19}H_{19}NO_4$
**FW**	325.35
**Temperature**	295
**Cryst. System**	triclinic
**Space group**	P-1
**Z**	2
**a (Å)**	4.9432(5)
**b (Å)**	13.7751(15)
**c (Å)**	13.8426(17)
**α (deg)**	117.355(12)
**β (deg)**	92.373(9)
**γ (deg)**	93.130(8)
**V ($Å^3$)**	833.58(18)
**D_{calc} (g/cm^3)**	1.296
**μ (mm^{-1})**	0.091
**Measd reflns**	5491
**Indep reflns**	2929
**Largest diff. peak/hole ($e/Å^3$)**	0.13/-0.15
**R_1[on F_0^2, $I>2\sigma(I)$]**	0.0493
**wR_2 (all data)**	0.1218

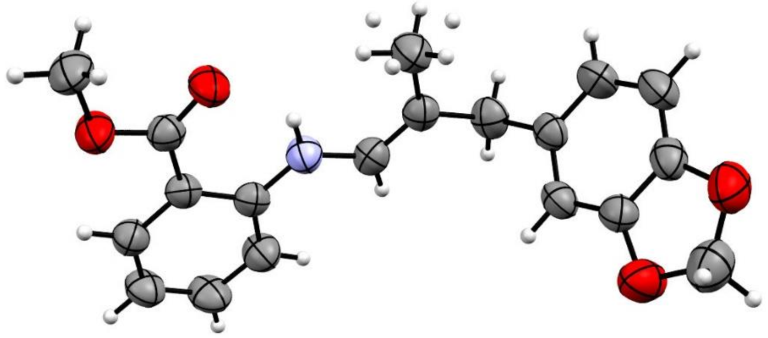

Figure S3. Ortep drawing of **SB2** (ellipsoids drawn at 50% probability).

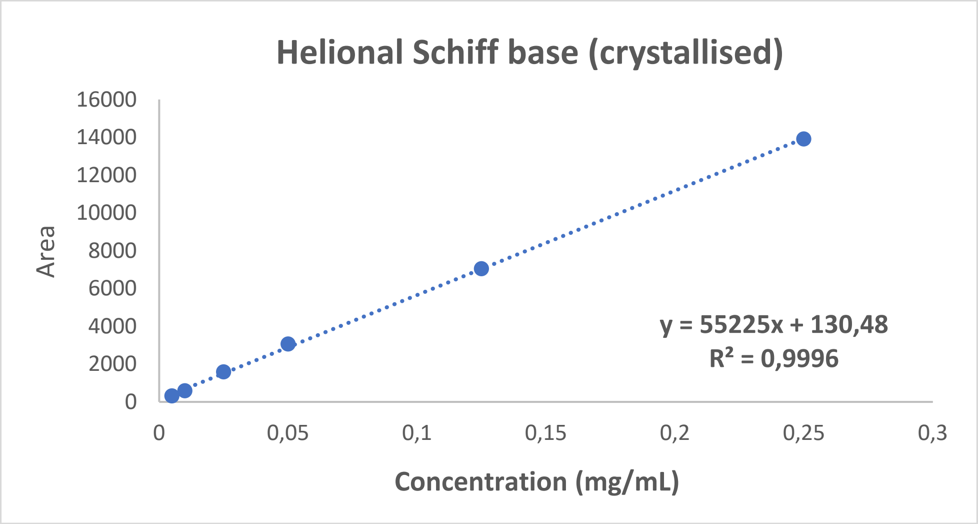

Figure S4. Calibration curve Helional Schiff base (crystallised).

The calibration curve was built starting from a solution of helional Schiff base crystals in ethanol. All the dilutions of this mother solution were injected in a HPLC-MS (DAD = 210 nm).

**Concentration**	**Area**
0,005	307,91562
0,01	580,49304
0,025	1577,84839
0,05	3059,14771
0,125	7035,26367
0,25	13901,7

Hydrolysis study in solution

After the reaction has undergone the optimum time for the specific Schiff base synthesis, the crude was dissolved in a 10 ml volumetric flask with the desired ratio of EtOH/H_2O. The concentration of unreacted MA at t_0 was determined from the calibration curve and, taking into account the dilution factor, the mg of unreacted MA were calculated. This quantity was subtracted from the initial mass of MA added for the reaction, obtaining the mass (and the moles) of reacted MA, and thus the starting moles of SB.

mmol of reacted MA = mmol of SB produced

$$Conversion\ (\%) = \frac{mmol\ of\ SB}{mmol\ of\ limiting\ reagent} \times 100$$

The hydrolysis of SB at each time (t_x) was calculated as:

$$Hydrolysis\ (\%) = \frac{mmol\ of\ SB\ at\ t0 - mmol\ of\ SB\ at\ tx}{mmol\ of\ SB\ at\ t0} \times 100$$

Hydrolysis study in gel (A or B)

The starting moles of SB were calculated right after the synthesis of each SB dissolving all the crude in EtOH and using the equations described in Method 1. The volume of the EtOH solution required to add 0.5% w/V concentration of SB (5 mg/mL) in each gel is calculated and added to the gelator solution as described in the Materials and Methods section.
At specific time intervals, each gel sample was completely dissolved in 1 mL of ACN, and to prepare the HPLC sample 200 µl of this solution were withdrawn and diluted to 1 ml of ACN. Considering the dilution factor, the hydrolysis calculation were done in the same way described above for the solutions.

Table S4. pH values for solutions and gels for all the SB

Media	Time (h)	SB1	SB2	SB2P	SB3	SB4
Sol. 85:15	0	5.92	5.71	4.81	5.66	5.93
Sol. 85:15	24	5.97	5.77	4.80	6.66	5.98
Sol. 70:30	0	5.03	5.14	4.83	5.27	5.69
Sol. 70:30	24	5.08	5.65	5.01	5.21	5.76
Sol. 70:30 + acid	0	3.55	3.62	3.60	3.57	3.74
Sol. 70:30 + acid	24	3.47	3.64	3.64	3.58	3.63
Gel A	0	5.62	5.19	6.58	5.55	5.78
Gel A	24	5.57	5.26	5.52	5.25	5.81
Gel B	0	3.86	4.10	3.81	3.75	4.04
Gel B	24	4.07	3.95	4.03	4.27	4.01

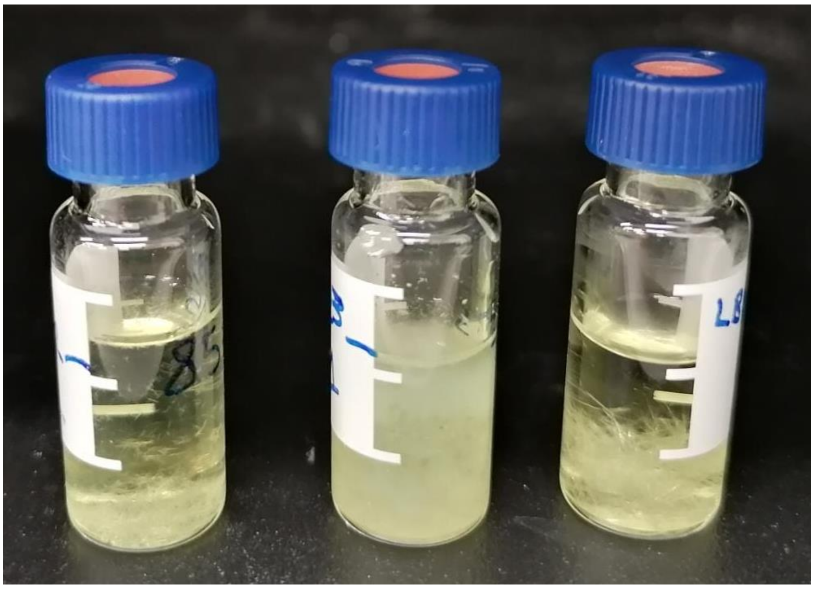

Figure S5. Photograph of **SB2P** solutions. From left to right: solution 85:15, solution 70:30, solution 70:30 and acetic acid.

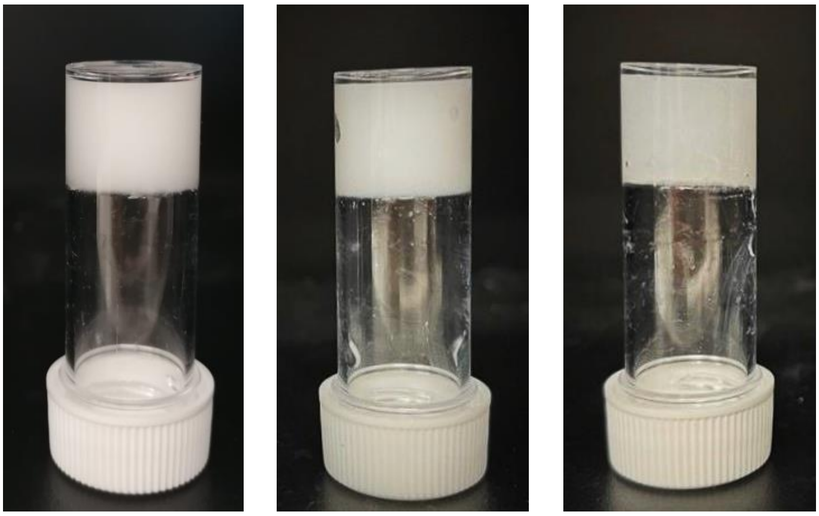

Figure S6. Photograph of the gels obtained with gelators **A** and **B**. From left to right: gelator **A** in 85:15 solvent ratio; gelator **A** in 70:30 solvent ratio; gelator **B** in 70:30 solvent ratio.

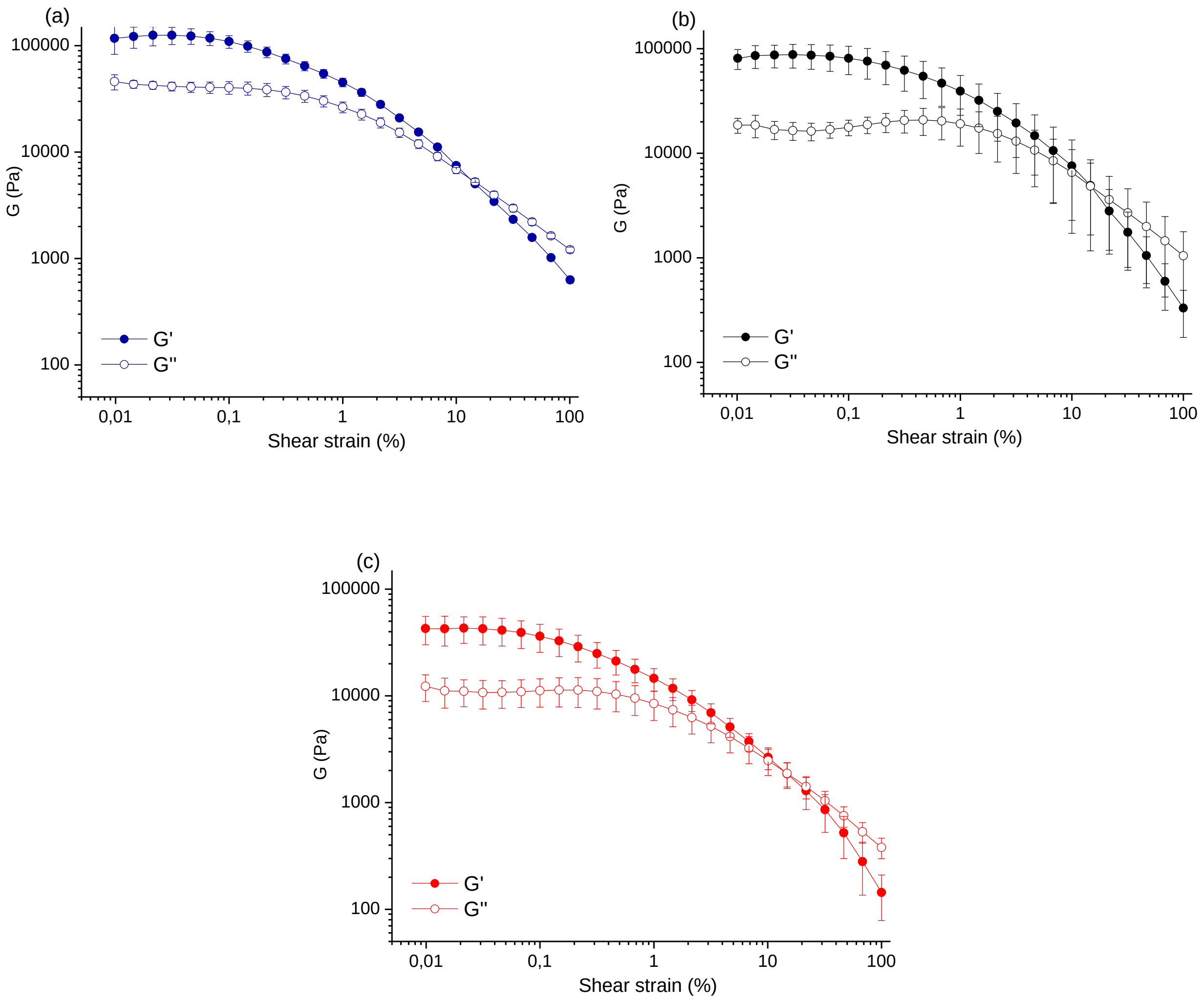

Figure S7. Amplitude sweep measurements for the gels prepared. (a) Gelator A (85:15); (b) gelator A (70:30), (c) gelator B (70:30). All measerements were repeated three times (mean and standard deviation values shown in the graph).

Table S5. Hydrolysis results for the SB under neutral conditions (mean values and standard deviations).

Time (h)	Conversion in ethanol/water solution (%)					Conversion in gel obtained from gelator A (%)				
	**SB1**	**SB2**	**SB2P**	**SB3**	**SB4**	**SB1**	**SB2**	**SB2P**	**SB3**	**SB4**
24	3.20± 1.10	5.55± 3.76	28.18± 7.00	40.14± 6.72	10.23± 6.47	0.25± 0.38	1.33± 0.77	30.13± 8.32	32.10± 5.60	5.33± 3.67
96	16.83± 7.08	11.28± 4.52	28.64± 2.05	42.77± 3.91	28.18± 7.01	6.92± 2.05	11.56± 6.9	34.18± 4.4	63.02± 6.65	9.68± 0.36

Table S6. Hydrolysis results for the SB under acidic conditions (mean values and standard deviations).

Time (h)	Conversion in ethanol/water solution (%)					Conversion in gel obtained from gelator B (%)				
	**SB1**	**SB2**	**SB2P**	**SB3**	**SB4**	**SB1**	**SB2**	**SB2P**	**SB3**	**SB4**
3	38.00± 2.10	44.58± 4.71	38.67± 13.59	70.36± 5.79	74.18± 3.02	23.44± 3.51	7.90± 2.55	33.77± 6.60	54.02± 15.09	45.83± 3.12
24	91.49± 4.34	74.91± 2.86	43.49± 8.83	72.73± 4.41	92.41± 1.19	67.57± 4.04	70.25± 2.70	49.36± 9.36	73.58± 4.21	84.77± 6.06
96	93.10± 1.86	95.25± 0.37	88.21± 9.73	89.17± 9.36	93.94± 2.1	82.95± 6.15	87.72± 3.70	61.80± 7.95	89.02± 4.69	85.33± 4.87
